# The Impact of Skip vs. Non-Skip N2 Lymph Node Metastasis on the Prognosis of Non-Small-Cell Lung Cancer: A Systematic Review and Meta-Analysis

**DOI:** 10.3389/fsurg.2021.749156

**Published:** 2021-10-12

**Authors:** Xinxin Wang, Haixie Guo, Quanteng Hu, Yongquan Ying, Baofu Chen

**Affiliations:** Department of Thoracic and Cardiovascular Surgery, Affiliated Taizhou Hospital of Wenzhou Medical University, Taizhou, China

**Keywords:** skip N2, NSCLC, non-skip N2, lymph node metastases, survival

## Abstract

**Objective:** The skip N2 metastases were frequent in non-small-cell lung cancer (NSCLC) and the better prognosis of NSCLC with a skip over non-skip N2 lymph node metastases is controversial. The primary aim of this study is to investigate the prognosis effect of skip N2 lymph node metastases on the survival of NSCLC.

**Setting:** A literature search was conducted in PubMed, EMBASE, and Cochrane Library with the term of “N2” or “mediastinal lymph node” or “mediastinal nodal metastases”, and “lung cancer” and “skip” or “skipping” in the title/abstract field. The primary outcomes of interests are 3- and 5-year survival in NSCLC.

**Participants:** Patients who underwent complete resection by lobectomy, bilobectomy, or pneumonectomy with systemic ipsilateral lymphadenectomy and were staged as pathologically N2 were included.

**Primary and Secondary Outcome Measures:** The 3- and 5-year survival of NSCLC was analyzed. The impact of publication year, number of patients, baseline mean age, gender, histology, adjuvant therapy, number of skip N2 stations, and survival analysis methods on the primary outcome were also analyzed.

**Results:** A total of 21 of 409 studies with 6,806 patients met the inclusion criteria and were finally included for the analysis. The skip N2 lymph node metastases NSCLC had a significantly better overall survival (OS) than the non-skip N2 NSCLC [hazard ratio (HR), 0.71; 95% CI, 0.62–0.82; *P* < 0.001; *I*^2^ = 40.4%]. The skip N2 lymph node metastases NSCLC had significantly higher 3- and 5-year survival rates than the non-skip N2 lymph node metastases NSCLC (OR, 0.75; 95% CI, 0.66–0.84; *P* < 0.001; *I*^2^ = 60%; and OR, 0.78; 95% CI, 0.71–0.86; *P* < 0.001; *I*^2^ = 67.1%, respectively).

**Conclusion:** This meta-analysis suggests that the prognosis of skip N2 lymph node metastases NSCLC is better than that of a non-skip N2 lymph node.

## Introduction

Non-small-cell lung cancer (NSCLC) is a leading cause of cancer-related death in the world (1). Approximately 30% of the NSCLC were locally advanced at the time of diagnosis (2). The most important risk factor in completely resected NSCLC is metastasis to the mediastinal lymph nodes (N2). The survival rates of N2-NSCLC after surgical treatment ranged from 19.2 to 40% which means that there was a large heterogeneity among N2-NSCLC (3–5). Skip metastases (pN0N2), pathological N2 NSCLC without hilar lymph nodes involvement (N1), were an important subgroup of N2-NSCLC. It was reported that skip N2 occurred in approximately 17.2–42.7% of surgically resected N2-NSCLC (6, 7). Skip N2 was reported to be an independent prognostic factor for better overall survival (OS) of the NSCLC (8, 9). However, the prognostic impact of skip N2 metastases was still under debate. The aim of this study was to elucidate the prognostic impact of skip N2 lymph node metastases on the OS of NSCLC through a meta-analysis of the published studies.

## Materials and Methods

### Search Strategy

A literature search was conducted in PubMed, EMBASE, and Cochrane Library from the date of database inception to Jan 2021. The following search terms were searched in the title/abstract field: “N2” or “mediastinal lymph node” or “mediastinal nodal metastases” and “lung cancer” and “skip” or “skipping”. Only articles in the English language were included. The reference lists of relevant review articles were checked to identify extra relevant articles.

### Inclusion and Exclusion Criteria

#### Inclusion Criteria

Studies must include patients with NSCLC who had undergone complete resection by lobectomy, bilobectomy, or pneumonectomy with systemic ipsilateral lymphadenectomy and were staged as pathological N2.

Studies must provide survival information on the patients with skip and non-skip N2 mediastinal nodal metastases.

#### Exclusion Criteria

Studies were excluded if the patients underwent the surgical operation of segmentectomy, wedge resection, or lymph node sampling.

Studies were excluded if the patients who were pathological N2 with pleural effusion or distant metastases.

Articles from the same study with duplicated data.

Studies do not provide survival information on patients with a skip or non-skip N2 mediastinal nodal metastases separately.

### Data Extraction

Data were extracted independently by two authors (XW, HG). If there was a disagreement, a consensus was achieved by discussion. The following data were extracted from each included article: first author, publication year, study design, inclusion criteria, number of patients, patients and tumor characteristics, operation techniques, survival curve, hazard ratio (HR), 3- and 5-year survival rates.

### Quality Assessment

The Newcastle–Ottawa Scale (NOS) was used to access the quality of included studies, with the highest score of 9. A high-quality study was defined as a study with a score of ≥6 **(****10****)**. The assessment was performed independently by two authors (QH and YY). If necessary, a third author (BC) was consulted to settle disagreements.

### Statistical Methods

Stata (version 12.0; StataCorp, College Station, Texas, United States) was used to perform all the statistical analyses. Between-study heterogeneity was calculated using Higgins' *I*^2^ statistics (11). A Mantel-Haenszel random-effects meta-analysis was performed for outcomes in consideration of interstudy heterogeneity. Studies with an *I*^2^ statistics of >50% were considered of a high degree of heterogeneity. A summary of the odds ratio (OR) and its corresponding 95% CI were computed for the dichotomous outcomes. To reduce the bias caused by different follow-up periods and the timing of censored patients between the two groups, OS in terms of log-transformed HR and 95% CI was analyzed using an inverse variance model. Relevant effect measures were calculated using methods described by Tierney et al. (12). Publication bias was assessed qualitatively using funnel plots and quantitively using Egger's linear regression method. Meta-regression analysis was conducted to evaluate the effects of covariates on the pooled estimates and the heterogeneity across studies with covariates including publication year, baseline mean age, the proportion of men, operation techniques, tumor location, and histological type. The overall effect was considered statistically significant if the two-sided *p-*value was < 0.05.

## Results

The literature search identified 409 relevant studies for review. Based on the title and abstracts, 60 studies were selected and reviewed for full text. Six studies were excluded for including patients with lymph node sampling. Nine studies were eliminated for the duplicated data. A total of five studies not providing survival information were excluded. Three studies including patients of not pathologically N2 were excluded. Five studies were eliminated because of including operation technique of segmentectomy or wedge resection. Lack of operation information was excluded in one study and three studies were eliminated for low quality scores. Three studies with patients of R1 or R2 resection were excluded. The full text was not available for four studies.

**Figure 1:**
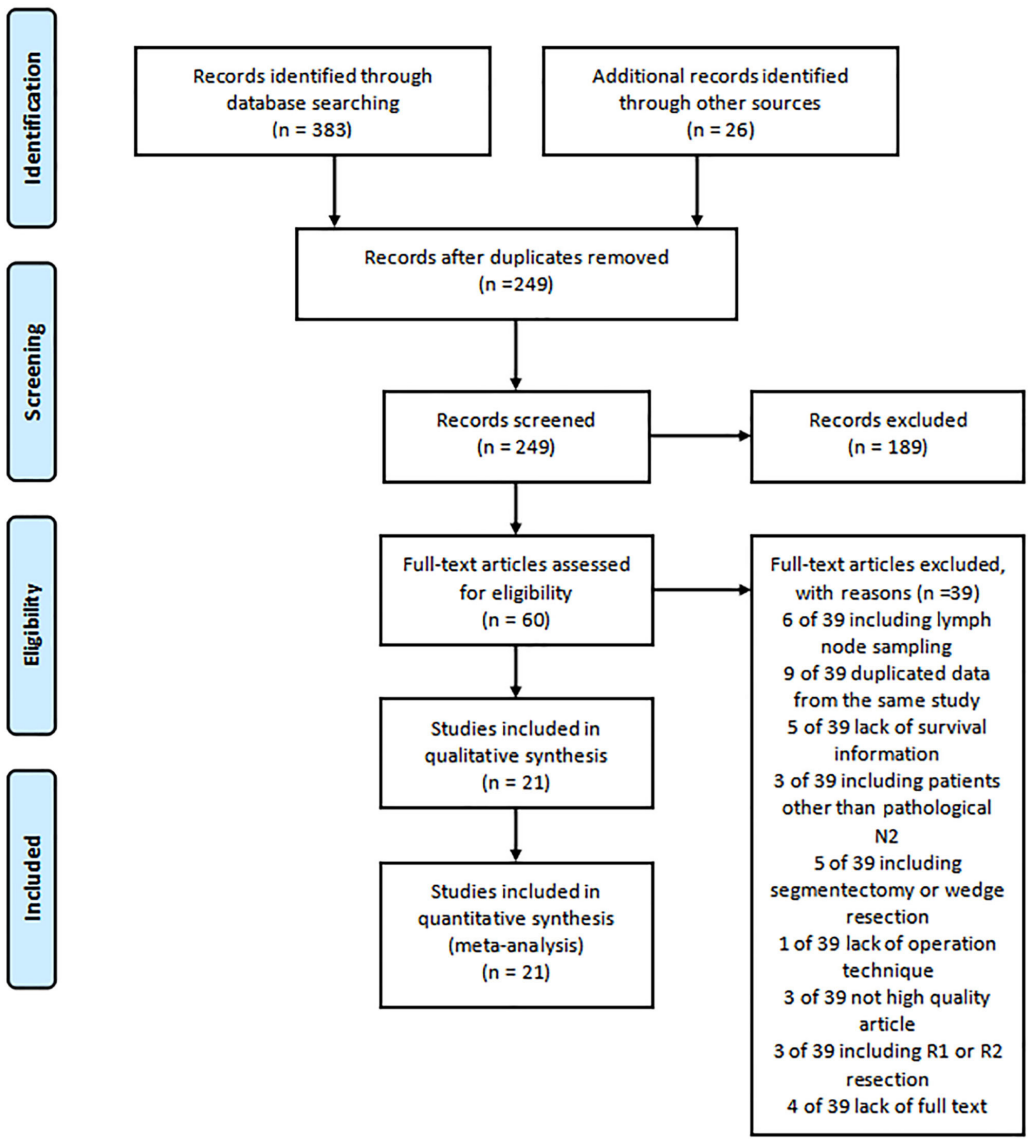
Flowchart of studies selection.

A total of 21 studies with 6,806 patients were included for the meta-analysis (Figure 1) (7, 9, 13–31). All the studies were retrospective studies. The clinical information of 2,258 patients of skip N2 and 4,548 patients of non-skip N2 were retrieved for further analysis. A total of 21 studies were performed in China (8), Japan (6), Italy (2), Germany (1), France (1), Turkey (1), Poland (1), and Croatia (1). All the studies were published between 1999 and 2020. Seven studies were published before 2010. Baseline characteristics in one study were adequately matched for age, gender, surgical procedure, tumor size, histology, T stage, and use of adjuvant therapy. The detailed baseline information was summarized in Table 1. The results of the quality assessment of the studies were shown in Table 2.

**Table 1:** Summary of baseline characteristics.

**Publication year**	**Country**	**Author**	**No. of Patients**		Mean age, Years^*^		**Male, %**		**Adjuvant treatment, %**		**Squamous cell cancer, %**		**Adenocarcinoma, %**		**Lobectomy, %**		**Bilobectomy, %**		**Pneumonectomy, %**		**Tumor location, left %**	
			**Skip N2**	**Non-skip N2**	**Skip N2**	**Non-skip N2**	**Skip N2**	**Non-skip N2**	**Skip N2**	**Non-skip N2**	**Skip N2**	**non–skip N2**	**Skip N2**	**non–skip N2**	**Skip N2**	**non–skip N2**	**Skip N2**	**non–skip N2**	**Skip N2**	**non–skip N2**	**Skip N2**	**non–skip N2**
2017	China	Jun Zhao et al. (13)	137	666	61 (32–78)	58 (30–81)	83.21	64.11	NR	NR	50.36	37.54	49.64	62.46	74.45	64.26	11.68	13.81	13.87	21.92	30.65	43.7
2019	China	Lin Wang et al. (14)	130	130	NR	NR	76.9	76.2	89.2	85.4	45.4	42.3	47.7	53.8	78.5	76.2	7.7	9.2	9.2	10	NR	NR
2018	Turkey	Serkan Yazgan et al. (15)	59	71	NR	NR	NR	NR	NR	NR	NR	NR	NR	NR	NR	NR	NR	NR	NR	NR	NR	NR
2007	Croatia	Nenad Ilic et al. (16)	21	64	NR	NR	62	81.3	NR	NR	46	21.9	18	62.5	71	57.8	0	12.5	29	29.7	38	43.8
2013	Japan	Makoto Sonobe et al. (17)	248	248	NR	NR	NR	NR	NR	NR	NR	NR	NR	NR	NR	NR	NR	NR	NR	NR	NR	NR
2001	Japan	Yukito Ichinose et al. (18)	110	296	NR	NR	NR	NR	NR	NR	NR	NR	NR	NR	NR	NR	0	0	NR	NR	NR	NR
2011	Japan	Tomoyuki Nakagiri et al. (19)	43	74	NR	NR	NR	NR	NR	NR	NR	NR	NR	NR	NR	NR	NR	NR	NR	NR	NR	NR
2015	China	Hang Li et al. (20)	45	132	56.9 ± 1.6	56.1 ± 1.4	44.44	57.58	100	100	0	0	100	100	NR	NR	0	0	NR	NR	33.33	53.03
2014	France	Marc Riquet et al. (21)	86	115	NR	NR	NR	NR	NR	NR	NR	NR	NR	NR	100	100	0	0	0	0	NR	NR
1999	Japan	Motoyasu Sagawa et al. (7)	76	102	NR	NR	NR	NR	NR	NR	NR	NR	NR	NR	NR	NR	NR	NR	NR	NR	NR	NR
2003	Poland	J. Gawrychowski et al. (22)	23	41	NR	NR	NR	NR	NR	NR	NR	NR	NR	NR	NR	NR	NR	NR	NR	NR	NR	NR
2003	Germany	Klaus L. Prenzel et al. (9)	17	28	68.1 (43–78)	59.5 (34–74)	70.6	71.4	NR	NR	53	32	35	61	NR	NR	NR	NR	NR	NR	58.8	53.6
2016	China	Dawei Guo et al. (23)	34	72	NR	NR	NR	NR	NR	NR	NR	NR	NR	NR	NR	NR	0	0	NR	NR	NR	NR
2020	China	J. Jin et al. (24)	98	199	NR	NR	68.4	71.4	100	100	NR	NR	57.1	53.8	98	96	0	0	2	4	32.7	41.2
2005	Italy	Christian Casali et al. (25)	63	120	NR	NR	NR	NR	100	100	NR	NR	NR	NR	NR	NR	NR	NR	NR	NR	NR	NR
2014	Japan	Junji Ichinose et al. (26)	25	42	NR	NR	NR	NR	NR	NR	NR	NR	NR	NR	NR	NR	NR	NR	NR	NR	NR	NR
2013	China	Dong Yan et al. (27)	34	81	NR	NR	NR	NR	100	100	NR	NR	NR	NR	NR	NR	0	0	NR	NR	NR	NR
2020	China	Lin Wang et al. (28)	81	206	NR	NR	NR	NR	NR	NR	0	0	100	100	NR	NR	NR	NR	NR	NR	NR	NR
2020	China	Xin Li et al. (29)	881	1772	59.66 ± 8.97	58.47 ± 8.98	66.9	62.7	42.2	42.8	33.4	23.9	60.6	69.3	83.5	82.8	NR	NR	11.7	14.2	49.2	55.9
2005	Japan	Masaki Tomita et al. (30)	25	35	NR	NR	NR	NR	NR	NR	NR	NR	NR	NR	NR	NR	0	0	NR	NR	NR	NR
2018	Italy	Pietro Bertoglio et al. (31)	22	54	66.4 ± 7.9	67.3 ± 7.3	81.8	68.5	86.4	94.4	27.3	48.1	63.6	44.4	100	92.6	0	3.7	0	3.7	22.7	48.1

* *Parenthesis indicates range; otherwise, data are expressed as mean ± SD. NR, not reported*.

**Table 2:** Quality assessment of the nonrandomized studies using the Newcastle–Ottawa Scale.

**Publicationyear**	**Study**	**Selection**				**Comparability (Based on design and analysis)**	**Outcome**			
		**Representativeness of exposed cohort**	**Selection of non-exposed cohort**	**Ascertainment of exposure**	**Outcome of interest absent at start of study**		**Assessment of outcome**	**Follow–up long enough for outcomes to occur**	**Adequacy of follow–up**	**Total score**
2017	Jun Zhao et al. (13)	1	1	1	1	0	1	1	1	7
2019	Lin Wang et al. (14)	1	1	1	1	2	1	1	1	9
2018	Serkan Yazgan et al. (15)	1	1	1	1	0	1	1	1	7
2007	Nenad Ilic et al. (16)	1	1	1	1	0	1	1	1	7
2013	Makoto Sonobe et al. (17)	1	1	1	1	0	1	1	0	6
2001	Yukito Ichinose et al. (18)	1	1	1	1	0	1	1	1	7
2011	Tomoyuki Nakagiri et al. (19)	1	1	1	1	0	1	1	0	6
2015	Hang Li et al. (20)	1	1	1	1	0	1	1	0	6
2014	Marc Riquet et al. (21)	1	1	1	1	0	1	1	0	6
1999	Motoyasu Sagawa et al. (7)	1	1	1	1	0	1	1	1	7
2003	J. Gawrychowski et al. (22)	1	1	1	1	0	1	1	1	7
2003	Klaus L. Prenzel et al. (9)	1	1	1	1	2	1	1	0	8
2016	Dawei Guo et al. (23)	1	1	1	1	0	1	1	1	7
2020	J. Jin et al. (24)	1	1	1	1	1	1	1	1	8
2005	Christian Casali et al. (25)	1	1	1	1	0	1	1	1	7
2014	Junji Ichinose et al. (26)	1	1	1	1	0	1	1	1	7
2013	Dong Yan et al. (27)	1	1	1	1	0	1	1	1	7
2020	Lin Wang et al. (28)	1	1	1	1	0	1	1	0	6
2020	Xin Li et al. (29)	1	1	1	1	0	1	1	0	6
2005	Masaki Tomita et al. (30)	1	1	1	1	0	1	1	1	7
2018	Pietro Bertoglio et al. (31)	0	0	1	1	2	1	1	0	6

### Overall Survival

**Figure 2:**
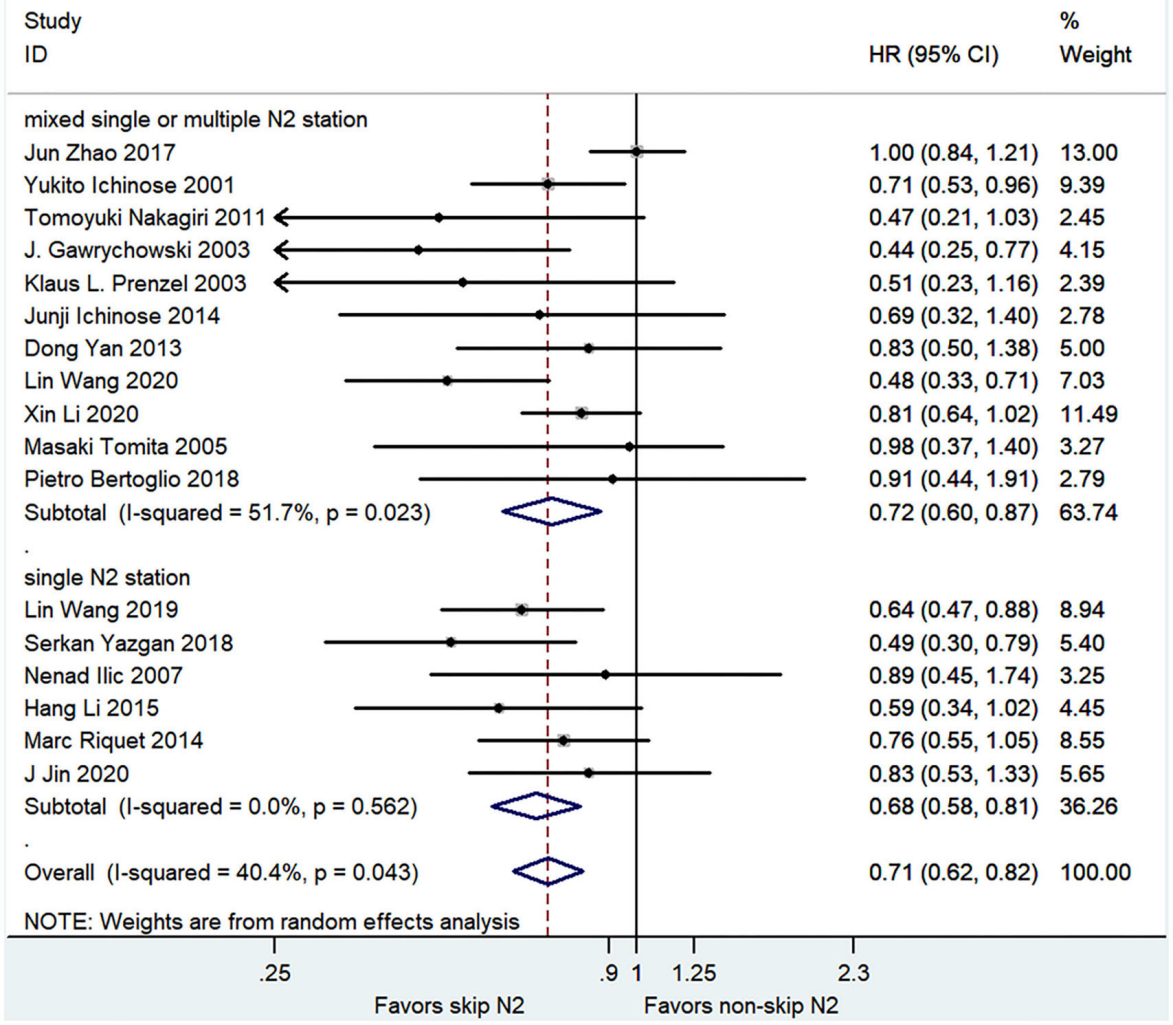
Pooled hazard ratio (HR) for overall survival (OS).

The pooled analysis demonstrated that the skip N2 group had a significantly better OS than the non-skip N2 group with a moderate heterogeneity (HR, 0.71; 95% CI, 0.62–0.82; *P* < 0.001; *I*^2^ = 40.4%; Figure 2). The skip N2 group still had a significantly better OS in the pooled analysis of multivariable analysis study (HR, 0.74; 95% CI, 0.65–0.86; *P* < 0.001; *I*^2^ = 0%; Table 3) and univariable analysis study (HR, 0.68; 95% CI, 0.55–0.84; *P* < 0.001; *I*^2^ = 56.8%; Table 3). The univariable studies had a high degree of heterogeneity. The skip N2 group had a significantly better OS in the pooled analysis of single skip N2 station and mixed single or multiple N2 station (HR, 0.68; 95% CI, 0.58–0.81; *P* < 0.001; *I*^2^ = 0% and HR, 0.72; 95% CI, 0.60–0.87; *P* < 0.01; *I*^2^ = 51.7%, respectively; Figure 2).

**Table 3:** Summary of sensitivity analysis for overall survival and 5-year survival rates.

**Variables**	**5-year survival rate**				**OS**			
	Data sets^*^	**Effect estimate** ^**†**^	***P* value**	**I^**2**^**	Data sets^*^	**Effect estimate** ^**†**^	***P* value**	**I^**2**^**
Adenocarcinoma	2 (464)	0.540 (0.416–0.702)	<0.001	0%	2 (464)	0.517 (0.376–0.711)	<0.001	0%
Mixed type	13 (3,100)	0.829 (0.765–0.898)	<0.001	48.6%	12 (4,981)	0.755 (0.671–0.895)	<0.01	31.5%
Publication year >2010	11 (2,959)	0.780 (0.683–0.891)	<0.001	75.4%	12 (5,183)	0.721 (0.614–0.845)	<0.001	48.1%
Publication year <2010	6 (936)	0.788 (0.719–0.863)	<0.001	0%	5 (660)	0.682 (0.529–0.878)	<0.01	13.2%
No. of patients >100	13 (3,650)	0.791 (0.711–0.879)	<0.001	73%	11 (5,446)	0.715 (0.611–0.836)	<0.001	52.9%
No. of patients <100	4 (245)	0.735 (0.622–0.867)	<0.001	0%	6 (397)	0.691 (0.520–0.918)	<0.05	2.3%
Mean age >60	4 (1,202)	0.842 (0.762–0.931)	<0.01	8.7%	2 (523)	0.678 (0.514–0.894)	<0.01	0%
Mean age <60	2 (253)	0.673 (0.497–0.911)	<0.05	13.5%	3 (2,906)	0.783 (0.640–0.958)	<0.05	0%
Adjuvant therapy for all	3 (595)	0.880 (0.772–1.003)	0.055	0%	2 (412)	0.835 (0.593–1.174)	0.299	0%
Adjuvant therapy not for all	14 (3,300)	0.757 (0.676–0.848)	<0.001	73%	15 (5,431)	0.696 (0.598–0.809)	<0.001	47.3%
Univariable analysis	9 (2,080)	0.715 (0.582–0.878)	<0.01	84%	11 (2,232)	0.681 (0.549–0.844)	<0.001	56.8%
Multivariable analysis	5 (958)	0.780 (0.703–0.866)	<0.001	0%	6 (3,611)	0.744 (0.645–0.859)	<0.001	0%
Single N2 station	5 (1,065)	0.738 (0.661–0.823)	<0.001	0%	6 (1,150)	0.682 (0.575–0.809)	<0.001	0%
Mixed single or multipleN2 station	12 (2,830)	0.808 (0.726–0.899)	<0.001	66.4%	11 (4,693)	0.722 (0.598–0.872)	<0.01	51.7%

* *Values in parentheses are the number of patients*.

† *Values are hazard ratios (HRs) 95% CIs for OS; HRs below 1 favor skip N2, whereas values are odds ratio (ORs) 95% CIs for 5-year survival rate; ORs below 1 favor skip N2*.

### 3- and 5-year Survival Rates

**Figure 3:**
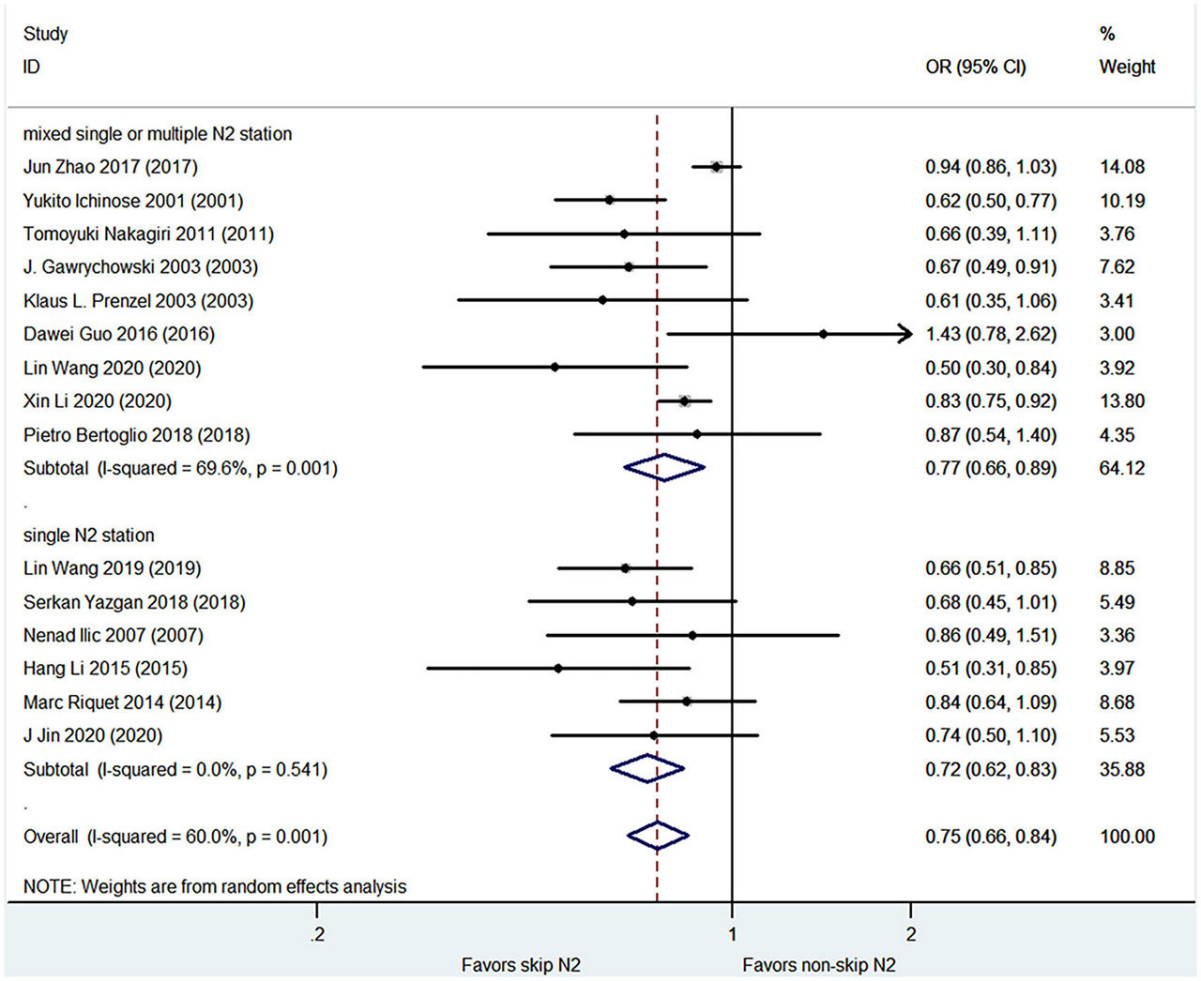
Pooled OR for 3-year survival rates.

**Figure 4:**
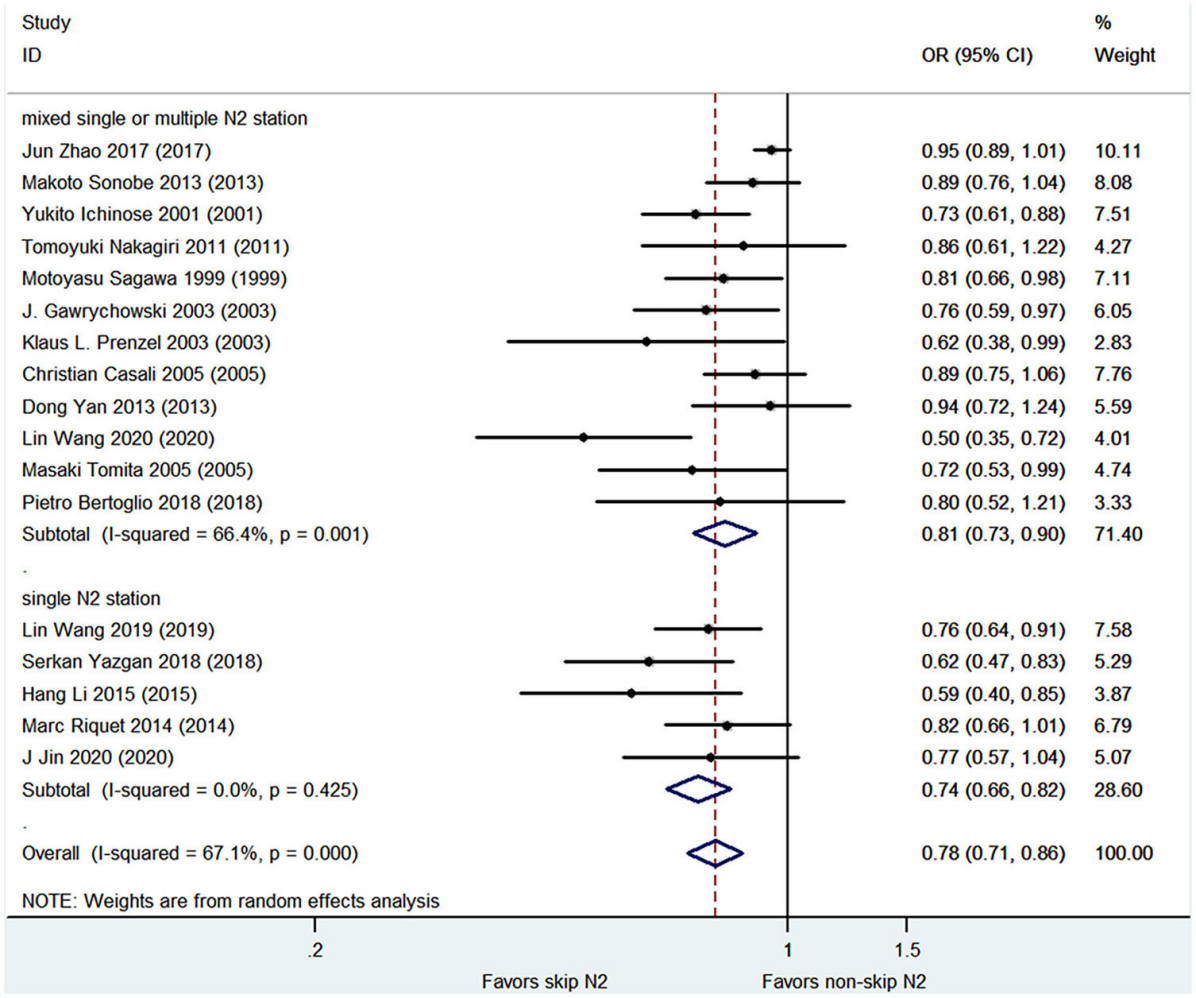
Pooled OR for 5-year survival rates.

The pooled analysis demonstrated that the skip N2 group had significantly higher 3- and 5-year survival rates than the non-skip N2 group (OR, 0.75; 95% CI, 0.66–0.84; *P* < 0.001; *I*^2^ = 60%; and OR, 0.78; 95% CI, 0.71–0.86; *P* < 0.001; *I*^2^ = 67.1%, respectively; Figures 3, 4) with a high heterogeneity. The mean 3- and 5-year survival rates in the skip N2 group were 60.4 and 43.4% and those of the non-skip N2 group were 46.6 and 25.3%, respectively.

### Sensitivity Analysis and Subgroup Analysis

The sensitivity analyses for OS and 5-year survival are summarized in Table 3. The sensitive analysis including publication year, number of patients, baseline mean age, the proportion of male, histology of adenocarcinoma or other, with or without adjuvant therapy, single or mixed single and multiple skip N2 station and analysis method of multivariable or univariable showed a survival benefit for skip N2 over non-skip N2, consistent with evidence from the primary outcome analysis except in the subgroup of adjuvant therapy.

### Meta-Regression Analysis

**Figure 5:**
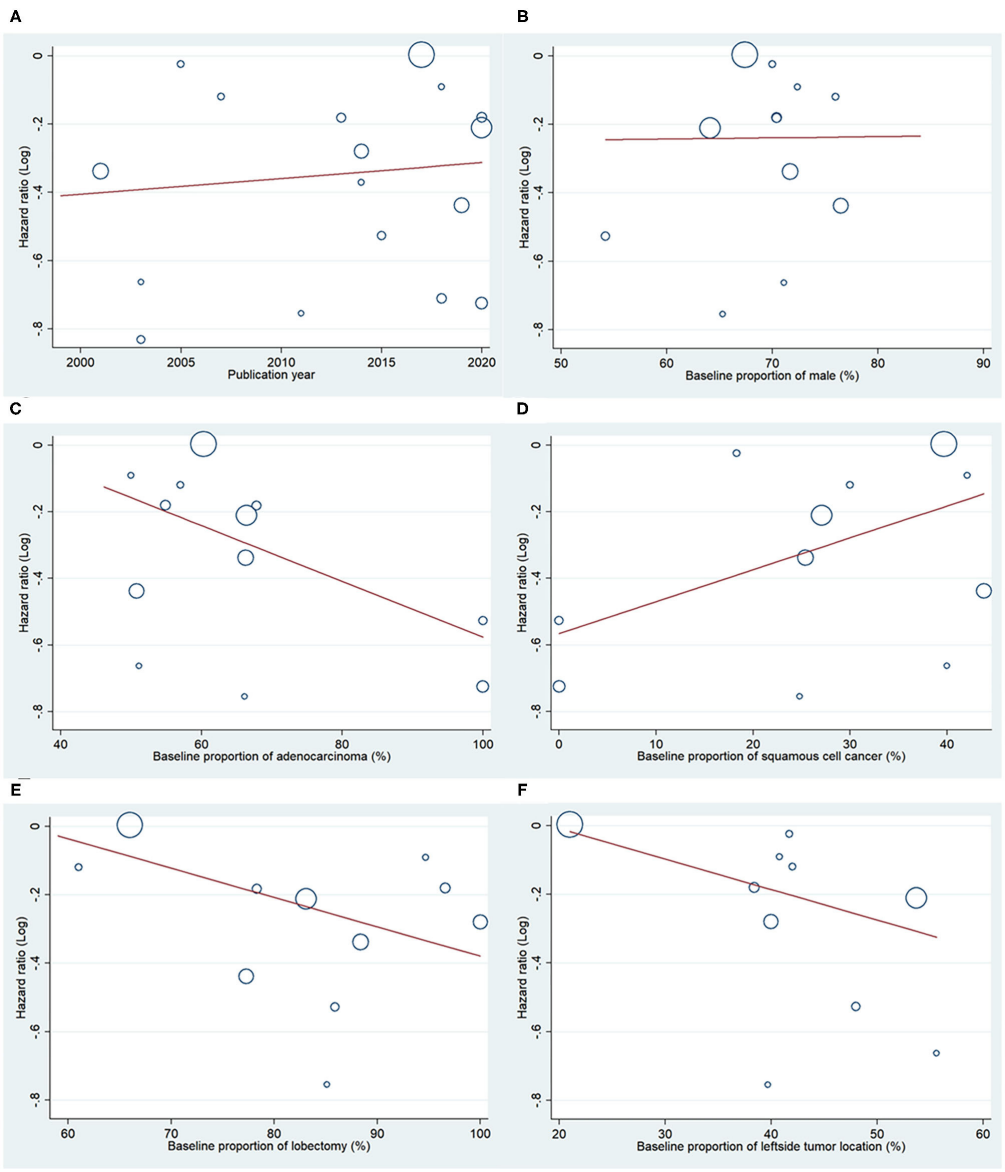
Meta regression analysis for OS. Bubble plot with fitted meta regression line of the log HR for **(A)** publication year, **(B)** baseline proportion of male, **(C)** baseline proportion of adenocarcinoma, **(D)** baseline proportion of squamous cell cancer, and **(E)** baseline proportion of lobectomy, **(F)** baseline proportion of the left side tumor location.

Meta-regression analysis showed a trend for publication year, baseline proportion of male, histology of adenocarcinoma or squamous cell, operation technique of lobectomy, tumor location of the left side. The trend was not statistically significant except for the histology of the squamous cells (*P* < 0.05) (Figure 5). The contribution of different study characteristics to the heterogeneity was calculated. The proportion of heterogeneity ranged from −22.67 to 100% for all the covariates. The remaining heterogeneity was small (τ2 range from 0 to 0.0316).

### Publication Bias

**Figure 6:**
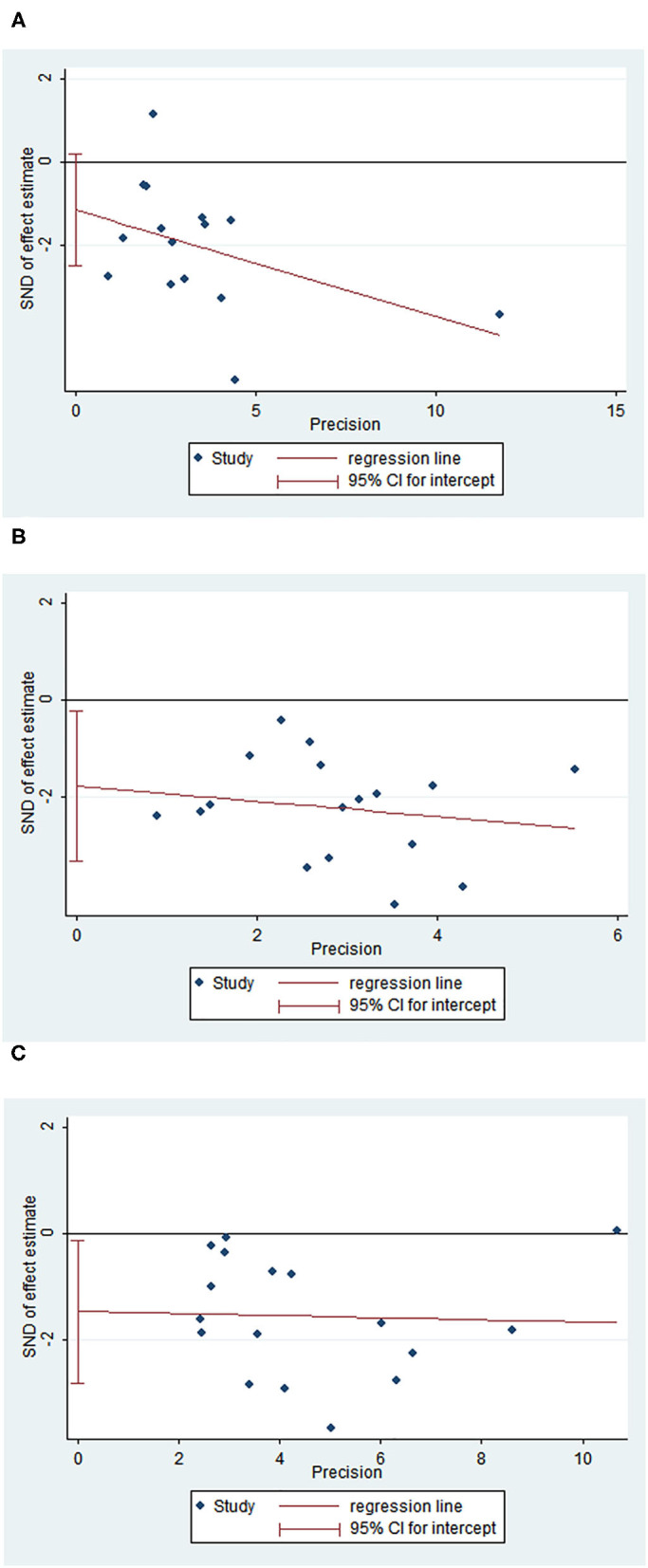
Funnel plot of OS, 3- and 5-year survival outcomes. **(A)** Egger plot for 3-year survival rates; **(B)** Egger plot for 5-year survival rates; **(C)** Egger plot for OS.

The funnel plots of OS and 3- and 5-year survival outcomes for skip and non-skip N2 groups are shown in Figure 6. There was publication bias for OS and 5-year survival outcomes (Egger's *p-*value, < 0.05) but no bias for 3-year survival outcome (Egger's *P* > 0.05).

## Discussion

Pathological N2 involvement is an important prognostic factor for NSCLC. Skip N2 disease is an important subclassification of N2 disease and accounts for 17.2–42.7% of resected N2 NSCLC (6, 7). The impact of skip N2 metastases on survival remains controversial. Several studies found a better OS for skip N2 disease than the non-skip N2 disease (17, 32). However, other studies did not find a significant difference in 5-year OS between the two groups (33, 34). Recently, the International Association for the Study of Lung Cancer suggested that the skip N2 should be treated as a new pN subclassification because of its better survival (35, 36). However, the evidence was based on the analysis of the patients from the cancer registry database and the clinical characteristics and operation details were not provided which still casts doubt on the conclusion. The present meta-analysis demonstrated that the skip N2 disease provided a better survival for NSCLC with a pooled analysis of the 24 studies.

The relationship is close in anatomy between N1 and N2 disease and the prognosis is also similar in some subgroups of N1 and N2 disease. It was reported that patients of N1 with hilar nodal involvement and patients of single N2 station metastasis had a comparable prognosis (37). Another recent study found that the 5-year OS was similar between patients with N1 metastasis and single skip N2 station metastasis, significantly better than the patients with single non-skip N2 station metastasis (15). Our study showed that the better prognostic impact is consistent in the single N2 station metastasis subgroup.

Multiple N2 stations involvement was reported to be a poor prognostic factor for survival (14, 31, 38). The new 8th tumor, node, metastasis (TNM) staging system for lung cancer did not further classify the N2b group into multiple skip N2 stations and multiple non-skip N2 station diseases. Our result showed that the influence of skip N2 in multistation metastases was not significant for OS although there were only two studies providing the survival information on the subgroup of N2 multistation metastases. Large studies may be needed to further elaborate the prognostic factor of multiskip N2 stations on survival.

Adenocarcinoma is an important histology subtype of NSCLC and the incidence has increased fast and accounted for almost one-half of all lung cancer in recent years (35, 39, 40). There was a higher risk of lymph node metastasis associated with adenocarcinoma compared with squamous cell cancer (41). Histological and molecular heterogeneity also existed in adenocarcinoma (42). The subgroup analysis of our study showed that skip N2 was also a benefit prognostic factor for adenocarcinoma.

The result of our study supported the 8th edition of the American Joint Committee on Cancer TNM staging system which classified the N2 into N2a1 (skip single station involved), N2a2 (non-skip single station involved), and N2b (multiple stations involved). Our result suggested that these patients with skip N2 metastases should be carefully recognized and surgery is an optimal option for these patients.

## Limitations

At first, this study is the first meta-analysis focusing on the prognosis of skip N2 lymph node metastasis on NSCLC and provides a convincing result that the prognosis of skip N2 lymph node metastasis NSCLC is better compared with that of non-skip N2 lymph node metastasis. Second, all the studies were retrospective studies. Third, the baseline clinical characteristics and adjuvant therapy in skip N2 and non-skip N2 were not matched among studies. Fourth, a high degree of heterogeneity and publication bias was found. Despite the use of meta-regression and numerous sensitivity analyses, significant heterogeneity remained. The results should be interpreted with caution. At last, most of the patients were Asians and the result may not be applicable for other ethnicities.

## Conclusion

The present meta-analysis suggests that the skip N2 lymph node metastases are a prognostic factor for better survival in N2-NSCLC.

## Data Availability Statement

The original contributions presented in the study are included in the article/supplementary material, further inquiries can be directed to the corresponding author.

## Author Contributions

XW drafted the manuscript. BC revised the manuscript. HG and XW extracted the data and XW did the analysis. QH and YY assessed the quality of the included studies. All authors contributed to the article and approved the submitted version.

## Funding

The work was supported by the Taizhou Municipal Science and Technology Bureau (No. 1801ky09) and the Medical Health Science and Technology Project of Zhejiang Provincial Health Commission (No. 2019KY773).

## Conflict of Interest

The authors declare that the research was conducted in the absence of any commercial or financial relationships that could be construed as a potential conflict of interest.

## Publisher's Note

All claims expressed in this article are solely those of the authors and do not necessarily represent those of their affiliated organizations, or those of the publisher, the editors and the reviewers. Any product that may be evaluated in this article, or claim that may be made by its manufacturer, is not guaranteed or endorsed by the publisher.
